# Interplay between Interfacial Energy, Contact Mechanics, and Capillary Forces in EGaIn Droplets

**DOI:** 10.1021/acsami.2c04043

**Published:** 2022-06-01

**Authors:** Shahrouz Amini, Xiaoping Chen, Jia Qing Isaiah Chua, Jinq Shi Tee, Christian A. Nijhuis, Ali Miserez

**Affiliations:** † Department of Biomaterials, Max Planck Institute of Colloids and Interfaces, 14476 Potsdam, Germany; ‡ Biological and Biomimetic Materials Laboratory, Center for Sustainable Materials (SusMat), School of Materials Science and Engineering, Nanyang Technological University (NTU), 50 Nanyang Avenue, Singapore 639798, Singapore; § Department of Chemistry and Environment Science, Fujian Province Key Laboratory of Modern Analytical Science and Separation Technology, Minnan Normal University, Zhangzhou 363000, China; ∥ Department of Chemistry, National University of Singapore, 3 Science Drive 3, Singapore 117543, Singapore; ⊥ Centre for Advanced 2D Materials and Graphene Research Centre, National University of Singapore, 6 Science Drive 2, Singapore 117546, Singapore; # Hybrid Materials for Opto-Electronics Group, Department of Molecules and Materials, MESA+ Institute for Nanotechnology and Molecules Centre, Faculty of Science and Technology, University of Twente, 7500 AE Enschede, The Netherlands; ∇ School of Biological Sciences, Nanyang Technological University (NTU), 60 Nanyang Drive, Singapore 637551, Singapore

**Keywords:** EGaIn, capillary bridge, depth-sensing nanoindentation, molecular junctions, self-assembled monolayers

## Abstract

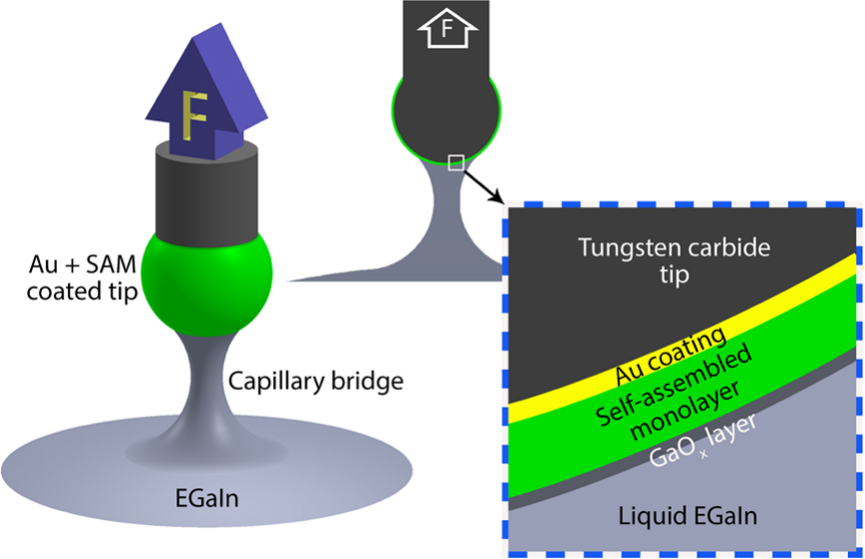

Eutectic gallium–indium (EGaIn) is increasingly employed as an interfacial conductor material in molecular electronics and wearable healthcare devices owing to its ability to be shaped at room temperature, conductivity, and mechanical stability. Despite this emerging usage, the mechanical and physical mechanisms governing EGaIn interactions with surrounding objects—mainly regulated by surface tension and interfacial adhesion—remain poorly understood. Here, using depth-sensing nanoindentation (DSN) on pristine EGaIn/GaO*_x_* surfaces, we uncover how changes in EGaIn/substrate interfacial energies regulate the adhesive and contact mechanic behaviors, notably the evolution of EGaIn capillary bridges with distinct capillary geometries and pressures. Varying the interfacial energy by subjecting EGaIn to different chemical environments and by functionalizing the tip with chemically distinct self-assembled monolayers (SAMs), we show that the adhesion forces between EGaIn and the solid substrate can be increased by up to 2 orders of magnitude, resulting in about a 60-fold increase in the elongation of capillary bridges. Our data reveal that by deploying molecular junctions with SAMs of different terminal groups, the trends of charge transport rates, the resistance of monolayers, and the contact interactions between EGaIn and monolayers from electrical characterizations are governed by the interfacial energies as well. This study provides a key understanding into the role of interfacial energy on geometrical characteristics of EGaIn capillary bridges, offering insights toward the fabrication of EGaIn junctions in a controlled fashion.

## Introduction

Due to the interesting non-Newtonian properties of eutectic gallium–indium (EGaIn) and its nontoxic properties, this liquid-metal alloy, among others, is widely employed in areas of research where it is important to have access to flexible/stretchable electrodes.^1−8^ This choice stems from the unique physicochemical properties of the EGaIn alloy: it is liquid at room temperature and spontaneously forms a thin oxide skin of gallium oxide (GaO*_x_*) under ambient atmospheric conditions that provide mechanical stability to the liquid core,^8−14^ allowing EGaIn to be readily manipulated into various shapes, such as cone-shaped tip electrodes (that are routinely used to measure charge transport phenomena across organic self-assembled monolayers (SAMs)^9,15−21^) and three-dimensional (3D) printed^6,22,23^ or stable structures in microchannels.^3,8,22^ This characteristic feature makes EGaIn a suitable material for additive printing,^6,23^ room-temperature microwelding/sintering,^24^ or applications in soft robotics.^25,26^

The GaO*_x_* layer that spontaneously forms on EGaIn is only about 0.7–3 nm thick;^27,28^ this thin oxide layer gives EGaIn its interesting non-Newtonian properties, but it is also a source of ambiguities. Arguably, these ambiguities are the most prevalent not only in (molecular) electronic applications but also in any other applications where it is important to understand how EGaIn interacts with surfaces: how smooth are the interfaces, what are the factors that contribute to the contact resistance, or how often can an EGaIn electrode be reused? For example, EGaIn is routinely shaped into cone-shaped tips, which, in turn, are used to form electrical contacts with the surfaces of SAMs on metal electrodes to obtain metal–SAM–GaO*_x_*/EGaIn junctions (a key element in molecular electronics).^15−21^ Here, the GaO*_x_* layer provides stability and prevents the bulk Ga-In alloy from alloying with the metal surface that supports the SAM, and it also is a source of uncertainty. The GaO*_x_*/EGaIn surfaces are rough (because of rupture of the GaO*_x_* during the formation of the tips), leading to high contact resistances (due to low effective contact areas).^21,29,30^ We also found that the cone-shaped EGaIn tips remained unchanged after 6–7 times of repeated indentations/contacts,^10^ but the origin of this “shape-memory” behavior is unclear. An explanation of all of these observations, however, remains elusive due to a lack of understanding of the indentation properties of EGaIn with its pristine GaO*_x_* at the molecular length scales.

Intuitively, how GaO*_x_*/EGaIn surfaces interact and flow across other surfaces (*e.g.*, SAMs, organic thin films, and stretchable materials) depends on the surface tension of those surfaces and the environment of EGaIn (which affects the formation of the GaO*_x_* layer). For example, it is well known that physisorbed water is important to consider at interfaces, but how the contact mechanics of EGaIn in air compare to those in water have not been studied. Especially for electronic applications, it is important to understand the wetting behavior of EGaIn as a function of the wetting properties of the target material or surface. For instance, in molecular electronics, it is well known that the molecule–electrode interaction plays a detrimental role in the conductance of metal–molecule–metal junctions.^31−33^ For instance, the contact resistance, *R*_C_ (in mΩ/cm^2^), of EGaIn with aliphatic SAMs with GaO*_x_* is 6 times higher than without the GaO*_x_* layer.^21^ Chen et al.^10^ and Wang et al.^37^ reported that *R*_C_ decreases by a factor of 4–6 for monolayers with different halide termini. These studies highlight the importance of improving our understanding of the differences in adhesion and wetting properties of EGaIn with different types of monolayers and, in extension, to other types of surfaces. In direct relation to these considerations, it is also important to consider the mechanical response of oxide skin/liquid-metal droplets during both compressive and tensile loading corresponding to the imposed stress regimes during electrode fabrication.

Here, we study the physical and mechanical interactions of EGaIn with its native GaO*_x_* layer with depth-sensing nanoindentation (DSN).^34,35^ This technique makes it possible to precisely measure contact forces and to investigate the deformation and reformation of GaO*_x_* under compressive and tensile loading. Indentation puncture and compression tests have been used to investigate the mechanical deformation of the EGaIn droplets.^28,36^ However, to the best of our knowledge, DSN using different tip surface chemistries and associated tip-EGaIn interfacial interactions has not been used to investigate the formation and geometry of the EGaIn capillary bridge. We indented EGaIn at various strain rates with ball-shaped tips coated with gold-functionalized SAMs of S(CH_2_)_11_*X* exhibiting vastly different interfacial energies (where *X* represents termini of CH_3_, NO_2_, NH_2_, or OH) in different environments (air and different aqueous environments). This approach gave us the following insights: (i) capillary bridges develop either positive or negative capillary pressures depending on the interfacial adhesive forces (important to understand for the fabrication of EGaIn tips or in 3D printing); (ii) specific chemical groups and the environment strongly affect the adhesive response of EGaIn; and (iii) the oxide layer repeatedly cracks and reforms during flow (explaining the shape-memory behavior of EGaIn) depending on the strain rate and environment. To demonstrate relevance, we tested these new insights on molecular junctions based on SAMs with different types of wetting behaviors from which we conclude that an increase in the adhesion force at the EGaIn/SAM interface results in a reduction of the contact resistance (and a concomitant increase in the quantum mechanical tunneling rates across these junctions). These new insights into the mechanical response of oxide skin/liquid droplets during both compressive and tensile loading under different chemical environments determine how EGaIn interacts with different types of surfaces and how those interactions affect the electrical response of not only molecular junctions but, in extension, also other types of electronic devices based on liquid metals (especially when repetitive movement or flow is involved typically encountered in flexible electronic devices or devices for healthcare).

## Results and Discussion

### Contact and Capillary Behavior of EGaIn under Different Chemical Environments

The mechanical response of EGaIn droplets and their flow behavior have been shown to be mainly defined by the presence and chemistry of an oxide layer.^3,8,14^ To further investigate the role of the oxide layer on the mechanical stability of EGaIn droplets, we adopted the “air-indent” DSN technique (see Methods) and designed three series of experiments, in which the chemistry of the oxide layer was altered by exposing the droplets to air (oxide layer), Milli-Q water (oxide monohydroxide), and 0.2 M NaOH (no oxide layer). We note that, recently, the adhesion behavior of the liquid Ga droplet/GaO*_x_* skin under axial loading was reported,^12^ but these measurements differ from ours in two ways. First, in our DSN configuration, the probe is rigid and indents a droplet of liquid metal, as opposed to having a probe made of liquid metal/oxide skin contacting the rigid substrate. Second, the axial displacement is simultaneously measured in our experiments, allowing us to directly detect a range of phenomena, such as localized fractures or the formation of capillary bridges as well as contact stresses on the oxide skin.

**Figure 1:**
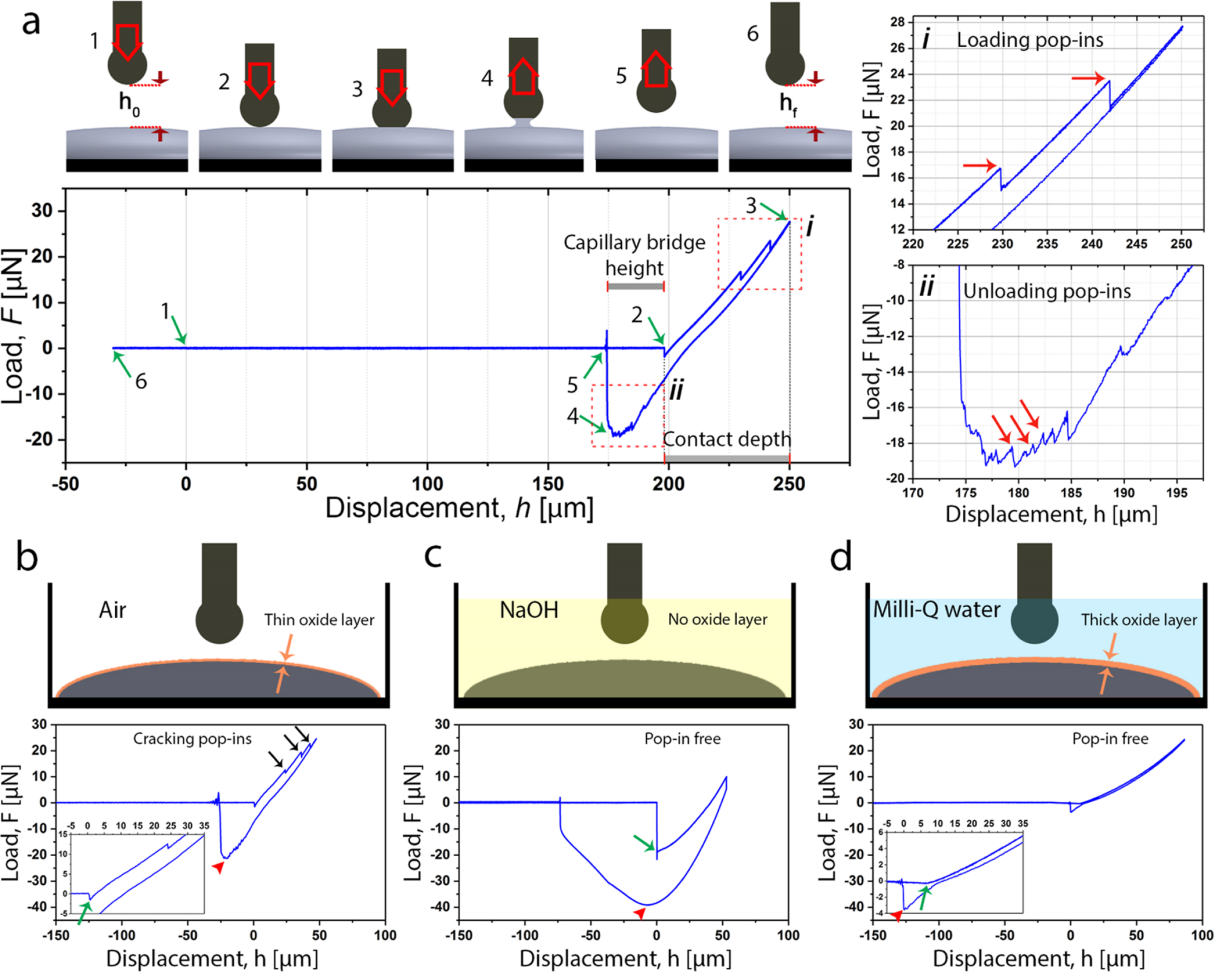
(a) Schematic of depth-sensing nanoindentation (DSN) of the EGaIn layer and representative load–displacement curve illustrating different steps of a loading/unloading cycle. The indentation process initiates away from the EGaIn surface (1) with no earlier surface approach, ensuring the interaction of the tip with a pristine surface with no damage/reformation on the oxide layer. Upon contact (2), a small attraction force is detected prior to compressive contact forces being generated by the moving tip contacting the surface of EGaIn. During loading, force instabilities are detected ((3), see also the top-right inset (i)), which can be attributed to oxide skin rupture and rapid reformation. At an imposed contact depth, the tip is retracted. Upon reaching the initial displacement (*h* = 0), the force does not return to zero. Instead, negative forces are detected, indicating the formation of a capillary bridge that connects the tip with EGaIn. Adhesion occurs until a maximum pull-off force ((4), see also middle-right inset (ii)) is reached, upon which the capillary bridge snaps and the force returns to 0 (5). Rupture and reformation of the GaO*_x_* skin are also detected during stretching of the capillary bridge (lower-right inset). (b–d) Loading/unloading cycles extracted from the samples exposed to air (b), 0.2 M NaOH (c), and water (d), showing the differentiation in the mechanical response of the samples due the variations in the oxide layer.

In air (Figure 1a,b), upon contact of the tip with the EGaIn surface, a small *F*_ad_ (*ca.* 1 μN) was initially detected, followed by contact (compressive) force (*F*) due to deformation of the oxide skin (note that in DSN, compressive contact forces are by definition positive and attraction and adhesive forces are negative). By further lowering of the tip, we detected drops in the force signal of *ca*. 1–2 μN, which can be attributed to localized cracking or rupture of the oxide skin beneath the tip (see the top-right panel in Figure 1a). During tip retraction, adhesive forces were detected, as evidenced by the negative force when the tip returned to its position at the initial contact point, which can be attributed to the formation of an EGaIn capillary bridge between the tip and the surface. The force eventually returned to zero once the EGaIn capillary bridge fully detached from the tip surface. It is noteworthy that irregular force jumps were also detected in the adhesive force regime (bottom-right panel of Figure 1a), *i.e.*, during elongation of the capillary bridge upon tip retraction. This has implications during fabrication of large-area tunnel junctions, and other types of interfaces involving EGaIn, since this behavior indicates that microfracture and regrowth of the oxide skin can occur not only during extrusion of EGaIn from the syringes or nozzles of 3D printers, for instance, but also during retraction during which the conical tip is shaped.^9,10^ A schematic illustration of the course of events during a full approach–loading–unloading cycle is summarized in Figure 1a.

We observed a significantly different indentation response when EGaIn droplets were immersed in 0.2 M NaOH (pH = 13.3), as shown in Figure 1c. NaOH was selected because it dissolves the oxide skin at a pH of >10,^14,37,38^ resulting in “skin-free” liquid EGaIn. In this case, a large “jump-in” instability occurred upon approach because of attraction forces between the liquid EGaIn and the spherical tip.^39^ As the imposed penetration depth increased, we observed a smooth loading curve without force instabilities, corroborating the absence of oxide skin in the alkaline environment. During tip retraction, we measured a large *F*_ad_ of *ca.* 40 μN as well as a large displacement before full detachment, indicating the formation of a large capillary bridge on the order of 60–70 μm in height. Large maximum adhesive forces during retraction are due to the enhanced stability of EGaIn capillary bridges, as discussed in the following sections.

Finally, we observed a third type of response when we submerged EGaIn in Milli-Q water prior to DSN measurements (Figure 1d). Under these conditions, the loading curve was continuous with almost no attractive force and initial jump-in, denoting a decrease in the EGaIn surface energy. Upon unloading, there was very minor curve hysteresis (thus indicating minimal viscoelastic loss) and the adhesion force was much weaker, with a pull-off force of only *ca.* 3 μN. Milli-Q water has been established to accelerate the kinetics of GaOOH skin formation,^40^ resulting in a weaker oxide skin.^41^ The shape of the loading/unloading indentation curve clearly indicates that the oxide skin is weak enough such that it does not rupture in a brittle manner during contact loading, as evidenced by the absence of loading pop-ins.

### Tip/EGaIn Interfacial Stability and Variations in Geometry of Capillary Bridges

**Figure 2:**
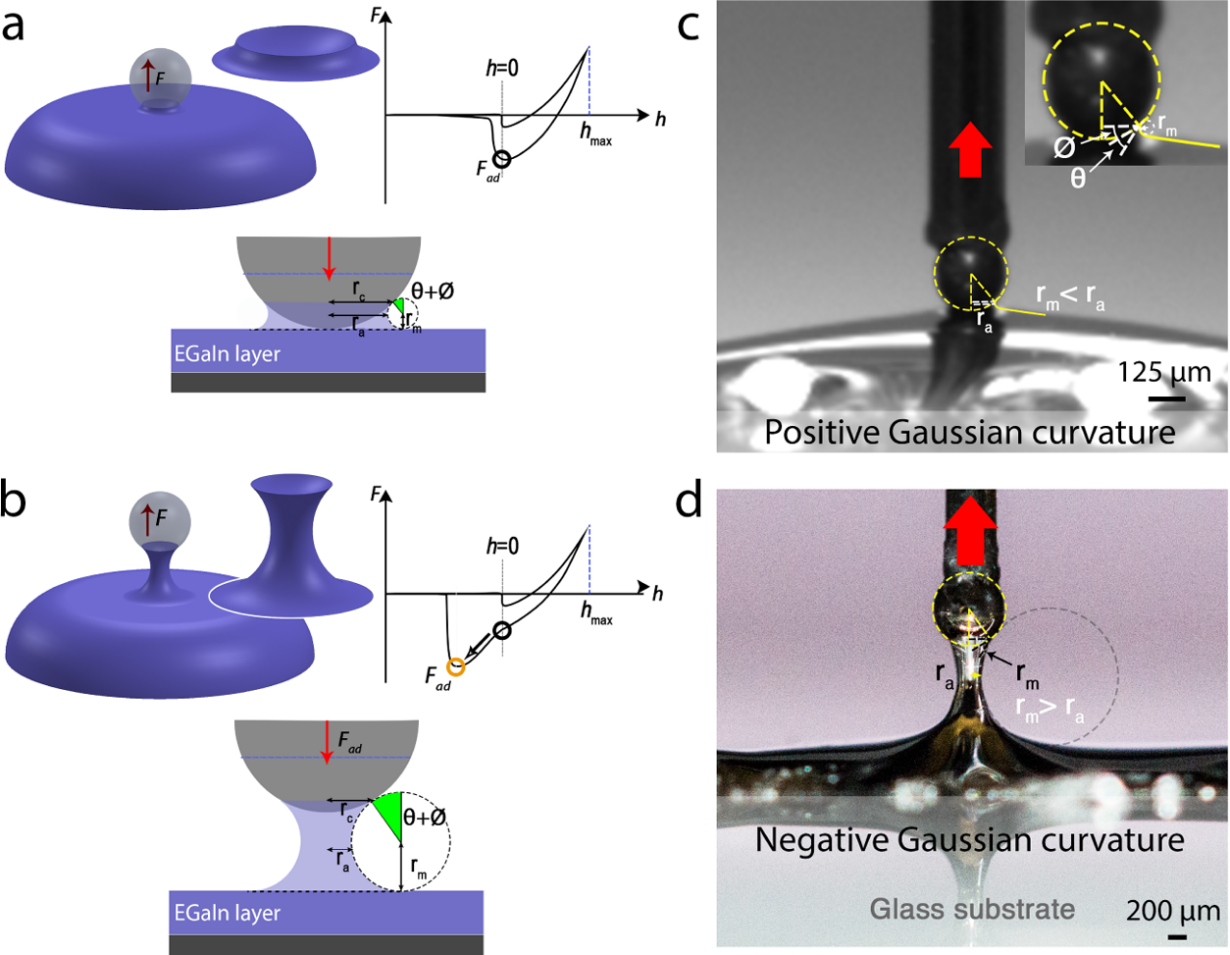
Two distinct contact regimes during DSN measurements of EGaIn samples. (a) In the first regime, the detachment of the EGaIn layer from the tip occurs roughly at the initial contact (*h* = 0) and *r*_m_ < *r*_a_. (b) In the second regime, the high interfacial adhesion between the tip and EGaIn triggers the development of large capillary bridges with *r*_m_ > *r*_a_. In these two regimes, the capillary bridge shape leads to either (c) positive or (d) negative capillary pressures, respectively, eventually resulting in repulsive or attractive forces during tip detachment from the EGaIn surface.

Based on the different micromechanical responses of the EGaIn capillary bridges described above, two distinct contact regimes can be identified, as schematically illustrated in Figure 2a,b. In the first regime (corresponding to the indentation response in air or water), the contact radius *r*_c_ and azimuthal radius *r*_a_ are always larger than the meridional radius of curvature of the EGaIn droplet *r*_m_ (*r*_c_ ≈ *r*_a_ > *r*_m_) and the maximum pull-off force occurs with a small (in air) or without (in water) a capillary bridge, *i.e.*, at a tip/surface displacement close to the initial contact (where *h =* 0). In the second regime (in NaOH), high adhesive forces between the tip and EGaIn surface result in the formation and development of large capillary bridges, whereby *r*_m_ > *r*_a_ during the tip/EGaIn separation. The total pull-off force (also indicated as *F*_ad_) at the tip/EGaIn layer interface during tip retraction can be written as the equilibrium between the surface tension force (*F*_γ_), the capillary pressure force (*F*_cp_), and the gravitational force (*F*_g_)^42^
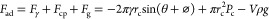
1where γ is the surface tension of EGaIn, (θ + ϕ) is the contact angle between the droplet and the vertical axis (see Figure 2), *V* is the volume of the EGaIn droplet, and *P*_c_ is the capillary pressure of the EGaIn droplet, *P*_c_ = _γ_(1/*r*_m_ – 1/*r*_a_), where *r*_a_ is the azimuthal radius of the EGaIn droplet or the capillary bridge at its center point and can be estimated as the contact radius *r*_c_ (*r*_a_*≈ r*_c_). Due to the small droplet volume, *F*_g_ is orders of magnitude smaller than the measured pull-off force and can be neglected (see the Experimental Section). In regime 1, since *r*_a_ > *r*_m_, the Gaussian curvature is positive (1/*r*_m_ – 1/*r*_a_ > 0), and the capillary pressure *P*_c_ exerts a repulsive force, which opposes the attractive force *F*_γ_. The total force *F* and the separation distance *h* are measured during DSN experiments, but the continuous values of *r*_a_ and *r*_m_ cannot be directly obtained. Therefore, we independently measured the θ and ϕ angles at the point of detachment (Figure 2c,d) and then plotted γ *vsr*_c_ for *r*_c_ values in the range of 80–120 μm, as shown in Figure S1 (corresponding to measured contact depths *h* < 50 μm; see Figure 1b). Using the mean value of *F*_ad_, we find that the surface tension γ of EGaIn in air ranges between 0.46 and 0.69 N/m. These values are in good agreement with those obtained using rheology (γ = 0.630 N/m)^8^ and a contact mechanic setup to measure larger droplets than in the current study (γ = 0.591 N/m).^12^ Using the same method, γ in water decreases to the range of 0.09–0.14 N/m, further emphasizing the important role of the oxide layer chemistry in decreasing the surface energy of EGaIn.

In the second regime, *r*_m_ > *r*_a_ at separation implies that the Gaussian curvature becomes negative (1/*r*_m_ – 1/*r*_a_ < 0) such that the capillary pressure now exerts an attractive force. This regime was observed for measurements carried out in NaOH where a large capillary bridge was measured (Figure 1c), but our setup was not suitable to measure the θ and ϕ angles. It also describes the behavior of Au-coated tips (Figure 2d) strongly adhering to EGaIn, triggering the formation of a capillary bridge upon tip retraction, as discussed later.

### Breaking and Reformation of the Oxide Layer at Different Strain Rates and Stress Regimes

**Figure 3:**
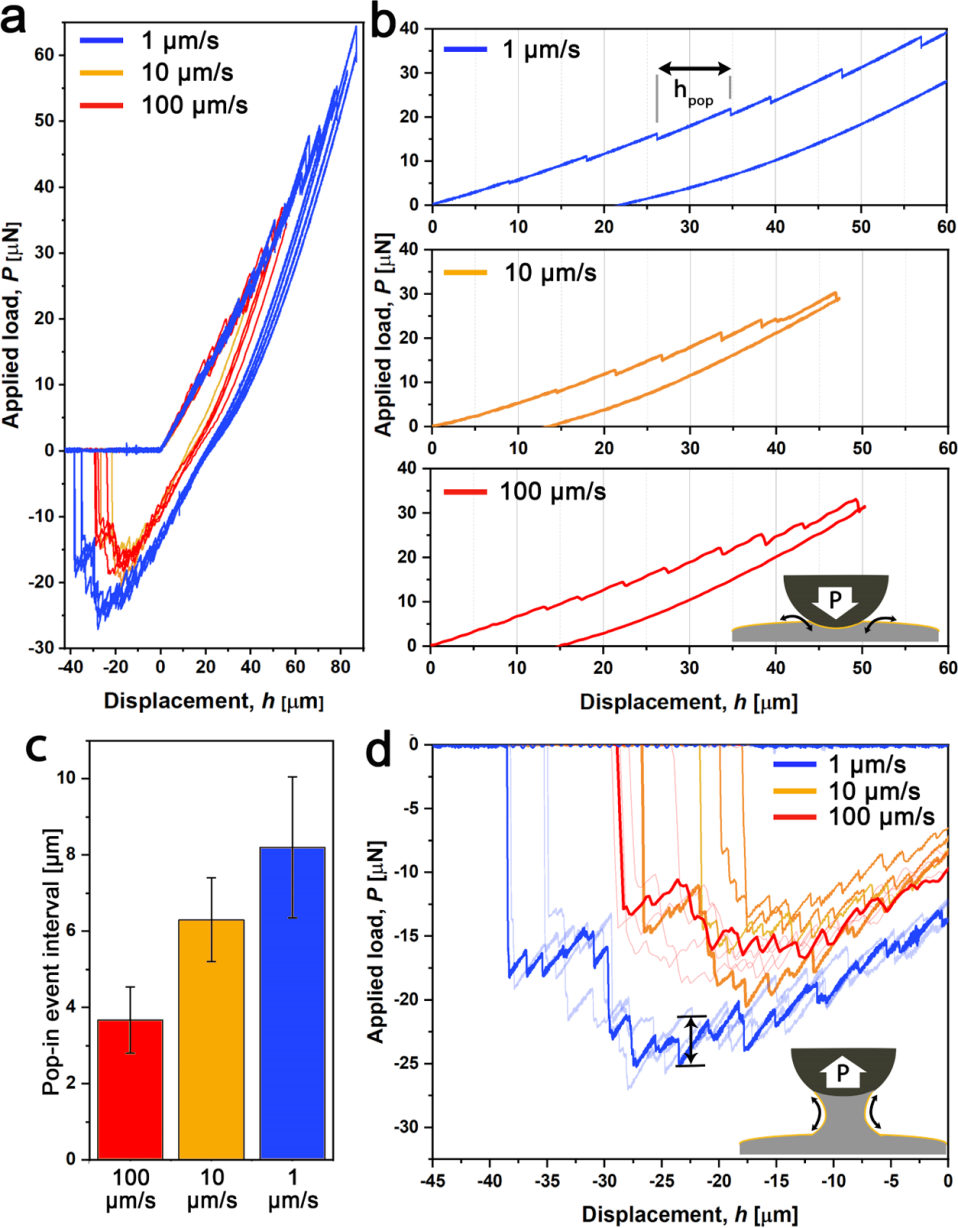
Deformation of the oxide layer during loading and unloading cycles at different strain rates. (a) DSN curves extracted at 1, 10, and 100 μm/s loading rates showing the presence of oxide-layer cracking behavior in both loading and unloading cycles. (b, c) Lower strain rates resulted in larger pop-in intervals, denoting that the cracking of the oxide layer can be delayed at lower strain rates. Data are mean ± sd (*n* = 20). (d) Similar behavior was detected during the unloading cycle (wherein EGaIn is predominantly subjected to tensile stress), but at lower strain rates, sharper decays in the adhesion forces were detected, indicating that the oxide layer can sustain higher tension strains at lower strain rates.

Rheological experiments^43^ have shown that the mechanical response of the EGaIn differs under different shear rates, but how the strain rate influences the behavior of the oxide layer is still unclear. We evaluated the deformation of the oxide layer at different strain rates (1, 10, and 100 μm/s) and stress regimes (compression/bending stresses during loading as well as tensile stresses during unloading) during our DSN measurements (Figure 3). We observed that at lower loading rates, the pop-in event interval size (*h*_pop_) increased, indicating that the oxide layer can sustain higher stresses under compression/bending loads at lower loading rates (Figure 3b,c). Similar sawtooth patterns related to breaking/reformation of the oxide layer were observed during the unloading cycle (wherein EGaIn is subjected to tensile stress), with larger force jumps detected at lower strain rates (Figure 3d). These observations suggest that despite a simultaneous recovery of the oxide layer, evidenced by repetitive cracking pop-in events, reformation time of the oxide layer plays a dominant role in the deformation kinetics of EGaIn under contact loads.

We attribute this trend to the following behavior: at lower strain rates, a thick oxide layer may be formed, and upon partial surface cracking of this layer (triggered by tensile stresses on the droplet), elastic energy is released. This release of elastic energy is higher for a thick than a thin oxide layer, resulting in pronounced force jumps until a new oxide layer is formed. This cycle of surface cracking/reformation of oxide layers then continues until the capillary bridge is fully separated from the surface. These repetitive cycles of cracking/reformation of oxide layers explain why EGaIn tips remain unchanged after repetitive contact formation of molecular monolayers as is routinely carried in molecular electronic applications (as mentioned in the Introduction).^10^ This “memory” effect of EGaIn with its GaO*_x_* layer is also important for soft electronic applications, where EGaIn is repetitively exposed to mechanical stress.

### Adhesion and Wetting of EGaIn on Different Types of SAMs

**Figure 4:**
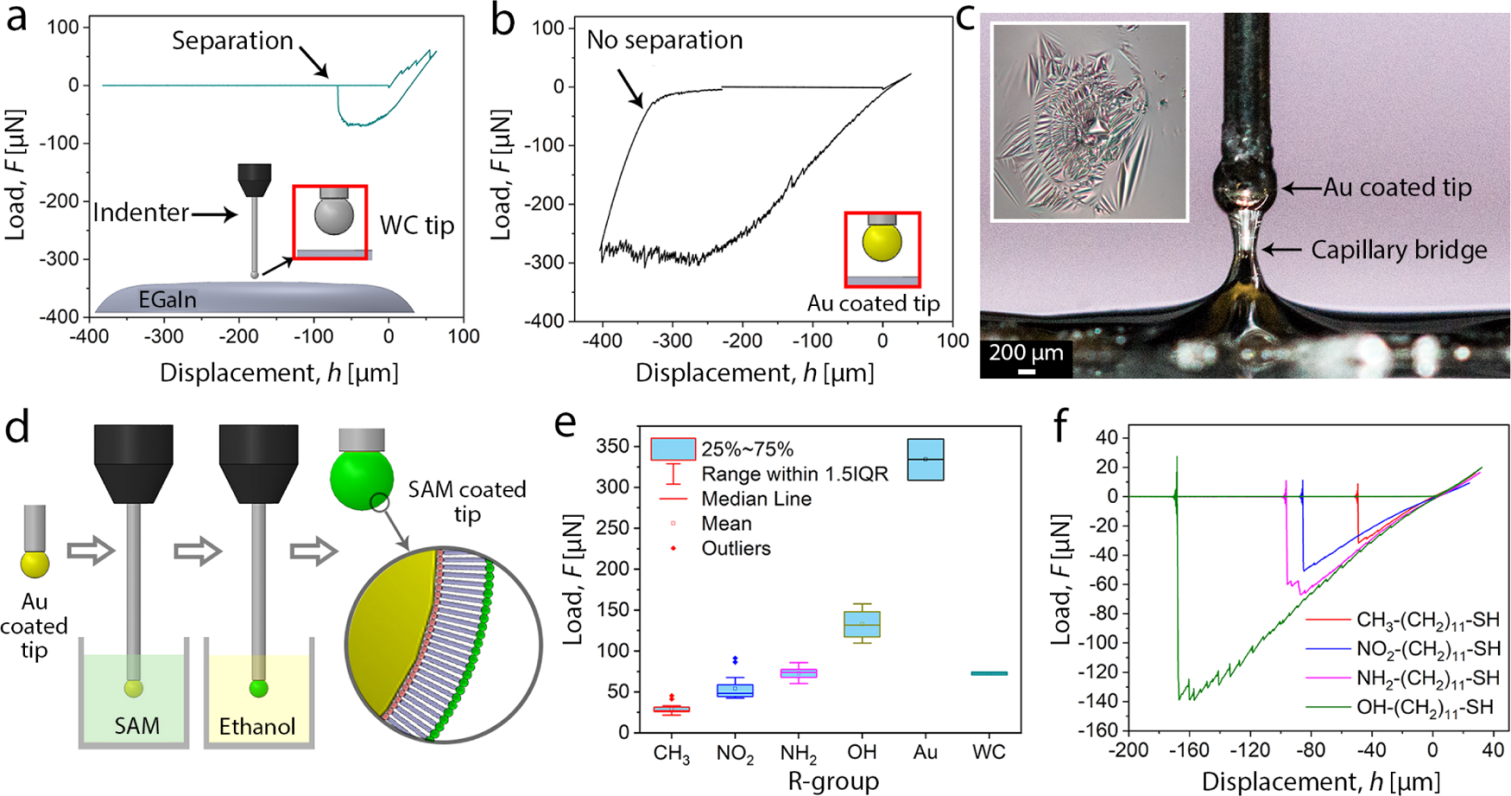
Adhesion behavior of EGaIn/self-assembled monolayers (SAMs). (a) Customized indentation tips were used to probe the wetting/adhesion behavior of EGaIn/SAMs interfaces. The curve shows a representative loading/unloading cycle of bare WC indenting EGain, with a pull-off force of *ca*. 75 μN. (b) Loading/unloading response for an Au-coated tip, showing much higher pull-off forces on the order of 300 μN and a capillary bridge height of 400 μm. (c) Side-view macrophotograph of a capillary bridge near maximum pull-off force and right before snapping. The inset shows a top-view of a probed surface after tip retraction and removal. The concentric patterns denote the formed crack patterns on the oxide layer. (d) Functionalization of the Au-coated tips with SAMs, resulting in SAM-functionalized indentation tips. (e) Mean adhesive (pull-off) forces of various SAMs/EGaIn interfaces. Data are mean ± sd (*n* ≥ 5). (f) Representative loading/unloading curves of SAMs/EGaIn illustrating their typical adhesion response.

As mentioned in the Introduction, it is important to study how EGaIn with its GaO_x_ layer interacts with surfaces that have different chemical functionalities and associated surface tensions. To this end, we prepared functionalized Au substrates with S(CH_2_)_11_*X* SAMs having different terminal *X* groups to measure how the SAMs influence the adhesive and contact mechanic responses of EGaIn/SAM interfaces. For these experiments, the tips were first Au-coated and then functionalized with SAMs derived from the following thiols: HS-(CH_2_)_11_-CH_3_; HS-(CH_2_)_11_-NO_2_; HS-(CH_2_)_11_-NH_2_; and HS-(CH_2_)_11_-OH (Figure 4d). The DSN experiments were then conducted as above using the “air-indent” mode to ensure access to a pristine surface of EGaIn. EGaIn forms noninvasive contacts with SAMs (*i.e*., it does not alter the SAM structure, as indicated by X-ray photoelectron spectroscopy).^44^ We repeated the measurements for at least 5 times, using new Au-coated tips with fresh SAMs for each set of loading/unloading cycle experiments. Bare tungsten carbide (WC) and Au-coated tips were also measured as control samples.

The range of *F*_ad_ values for the different types of SAMs could be grouped into three different categories, as depicted in Figure 4e,f. The SAMs derived from HS-(CH_2_)_11_-CH_3_ yielded the smallest pull-off force with a mean value of *F*_ad_ = 29 ± 6 μN. The pull-off adhesions of HS-(CH_2_)_11_-NO_2_ and HS-(CH_2_)_11_-NH_2_ were similar, with intermediate mean values of 54 ± 13 and 73 ± 7 μN, respectively. Finally, the largest pull-off forces were measured for SAMs derived from HS-(CH_2_)_11_-OH with *F*_ad_ = 133 ±20 μN. The bare Au-coated tip on EGaIn exhibited larger pull-off forces than all SAMs with *F*_ad_ = 334 ± 25 μN.

For measurements with large pull-off forces (Au-coated tips and -OH-terminated SAMs), we note that a long capillary bridge formed before complete rupture occurred. In such cases, strong interfacial adhesion induces elongation of the EGaIn droplet into a connecting bridge until the Gaussian curvature becomes negative (1/*r*_m_ – 1/*r*_a_ < 0, Figure 2d). According to eq 1, this increases the total adhesion between the EGaIn droplet and the substrate, thereby stabilizing the capillary bridge until snapping and separation occur. This insight is important for the fabrication of EGaIn tips, which are sometimes hampered by the formation of long capillary bridges.

It is important to mention that the representative curves shown in Figure 4f were reproducible for a restricted number of loading/unloading cycles. After a few cycles, and in some cases as soon as the second loading/reloading occurs, much larger pull-off forces (*F*_ad_) approaching that of the Au-coated tip were measured, which we attribute to alloying of the EGaIn with the Au layer likely due to cycling fatigue related to defects in the SAM. This fatigue suggests that EGaIn top electrodes can only be reused for a limited number of times. For EGaIn applied in molecular electronics, normally the cone-shaped tip electrodes are only 3–6 times reused for empirical reasons (*i.e*., to maximize yield and reproducibility between experiments).^10,15,29,45^ Based on the results reported here, we conclude that mechanical fatigue and possible damage to the SAMs by repetitive indentation with EGaIn are effectively reduced when one tip is often used 3–6 times.

### Electrical Characterizations of Molecular Junctions and Contact Angle Measurements

We used conical EGaIn top electrodes to form the molecular junctions in the form of Ag-S(CH_2_)_11_*X*//GaO*_x_*/EGaIn (“-” represents covalent contact, “//” represents noncovalent contact, and “/” gives the interface between the GaO*_x_* and bulk EGaIn) and to measure the *J*(V) (*J* = current density in A/cm^2^ and *V* = applied bias voltage in V) characteristics of the junctions following previously reported methods.^45^ Briefly, for each type of SAM on Ag bottom electrodes, we formed ∼20 junctions for each of which we recorded 20 *J*(V) traces by applying bias to the EGaIn top electrode (the Ag electrode was connected to the ground), which we used to determine the Gaussian average of the logarithm of the absolute current density *J*, <log_10_|*J*|>_G_, along with the Gaussian log-standard deviations (σ_log,G_); see the Experimental Section, Figures S2–S4, and Table S1 for experimental details and results. We also conducted impedance spectroscopy using 30 mV of sinusoidal perturbation at the frequency range of 10^2^–10^6^ Hz with 10 points per decade for the junctions to separate the resistance of the SAM (*R*_SAM_, in Ω·cm^2^) and the contact resistance (*R*_C_, in mΩ·cm^2^), which is dominated by the EGaIn–SAM contact, following previous methods (see details in the Experimental Section, Figures S5–S7 for the residual plots, the Bode, Nyquist, and phase angle *vs* frequency plots of the molecular junctions, and Table S2 for the impedance fitting results).^46,47^

**Figure 5:**
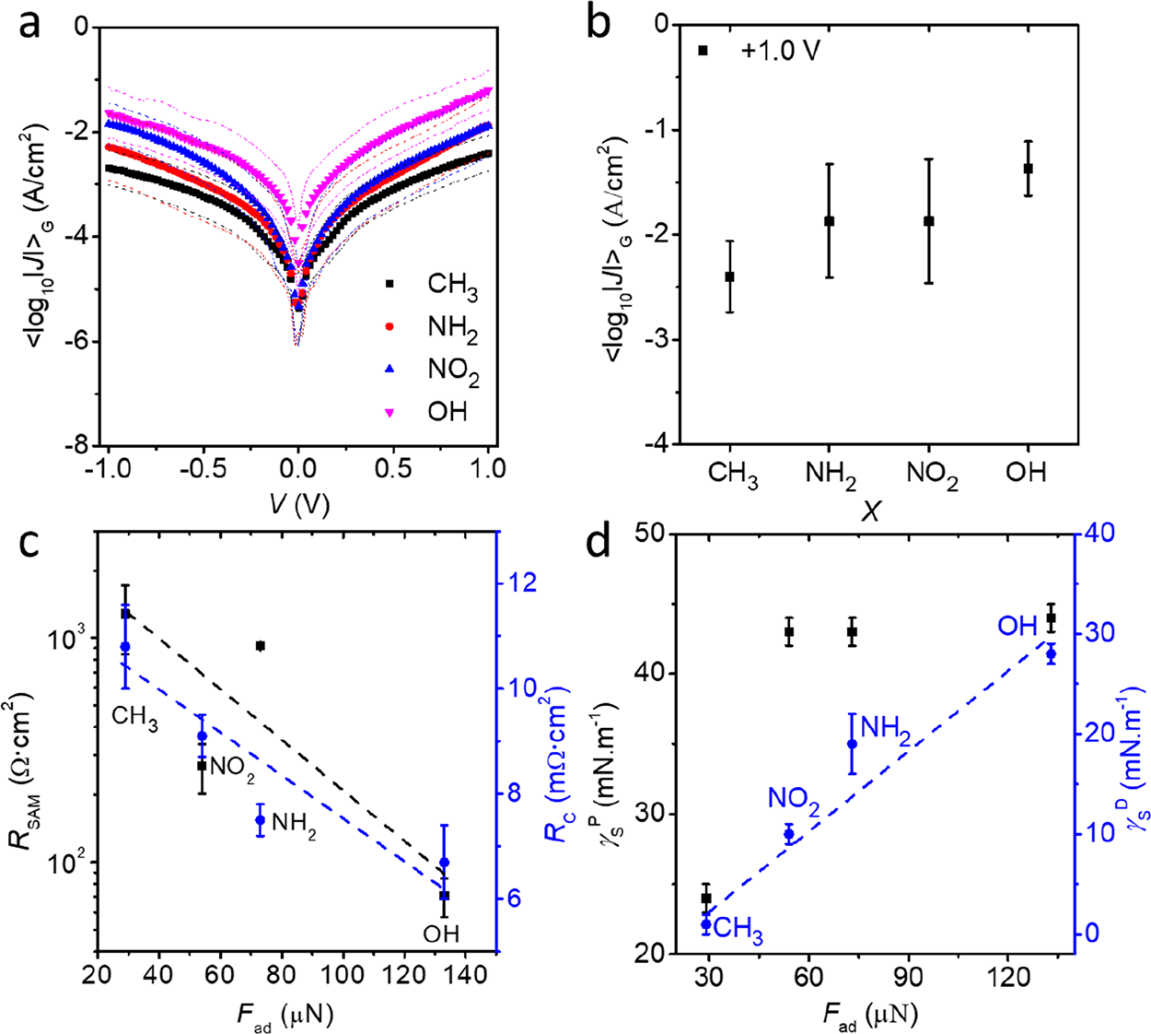
Electrical results of the Ag-S(CH_2_)_*n*_*X*//GaO*_x_*/EGaIn junctions. (a) Gaussian log-averaged current density (<log_10_|*J*|>_G_) *vs* applied bias of V for Ag-S(CH_2_)_*n*_*X*//GaO*_x_*/EGaIn junctions. Black squares are for the <log_10_|*J*|>_G_ of *X* = CH_3_, red circles are for *X* = NH_2_, blue up triangles are for *X* = NO_2_, purple down triangles are for *X* = OH, and green diamonds are for *X* = COOH. The colored dots are Gaussian log-standard deviation of σ_log,G_. (b) <log_10_|*J*|>_G_ at an applied bias of ±1.0 V for different terminal groups. The error bars are σ_log,G_. (c) *R*_SAM_ and *R*_C_*vsF*_ad_ from junctions of different terminals. The values of *R*_SAM_ and *R*_C_ are represented by black squares and blue circles, respectively. (d) Polar (black squares) and dispersive portions (blue circles) of the surface free energy *vsF*_ad_ of SAMs on Ag from contact angle measurements. The error bars in panels (c, d) represent the standard deviations from three different measurements. The dashed lines are guides to the eye.

Figure 5a shows the values of <log_10_|*J*|>_G_ plotted against *V* for the Ag-S(CH_2_)_11_*X*//GaO*_x_*/EGaIn junctions. Figure 5b shows that the value of *J* increases by ∼1.0 order of magnitude when *X* = H was changed to *X* = OH following the order OH > NO_2_ > NH_2_ > CH_3_. The trend of *R*_SAM_ (Figure 5c) agrees with the changes in the values of *J* (Figure 5a) following the order CH_3_ > NH_2_ > NO_2_ > OH. As we have reported before, *R*_C_ is dominated by the SAM//EGaIn contact resistance and not by the Ag-SAM resistance. Figure 5c shows a clear correlation between *R*_C_ and *F*_ad_: *R*_C_ decreases with increasing values of *F*_ad_. These results imply that OH termini have the strongest interaction with the GaO*_x_* and CH_3_ the weakest. In general, strong molecule–electrode interactions lead to large molecule–electrode coupling parameters (*i.e.*, following the well-known Landauer–Büttiker approach; see the Supporting Information) and large current densities (and low resistances). In other words, the observed trends in *F*_ad_, *J*, *R*_C_, and *R*_SAM_ are in excellent agreement and can be all explained by changes in SAM//GaO*_x_*/EGaIn interactions.

To complement the DSN measurements and to explain the correlation between *F*_ad_ and *R*_C_ in more detail, we measured the surface energy of the SAMs using static contact angle measurements using liquids of H_2_O and CH_2_I_2_. We analyzed our results with the OWRK model (OWRK stands for Owens, Wendt, Rabel, and Kaelble)^48−50^ (see the Experimental Section, Figures S8-12, and Tables S3-4) to extract the dispersive γ_s_^D^ and the polar γ_s_^P^ components of the surface free energy γ_s_. Figure 5d shows the plot of γ_s_^D^ and γ_s_^P^ as a function of *F*_ad_ for all *X*. This plot shows that γ_s_^D^, in contrast to γ_s_^P^, correlates very well with *F*_ad_. Hence, we conclude γ_s_^D^ of the SAM does affect the interaction with the GaO*_x_*/EGaIn surface, which, in turn, affects the electronic properties, especially *R*_C_ (and consequently *R*_SAM_). This conclusion also agrees with our earlier observations where we suggested a correlation between measured charge transport rates and the adhesion strength of the SAM//GaO*_x_*/EGaIn interface.^17,47,51^ For the sake of completion, we also measured the interfacial energy γ*_i_* between the SAMs and GaO*_x_*/EGaIn from static contact angle values of GaO*_x_*/EGaIn droplets on the respective SAMs (Figure S13). Figures S14 shows that γ*_i_* also correlates well with *F*_ad_. This correlation between wetting and adhesion is expected^39^ since a smaller contact angle (higher interfacial energy) will result in a larger area of SAMs covered by EGaIn, which translates into increased adhesive forces during pulling of the capillary bridge.

## Conclusions

Our results establish that DSN is a versatile tool to characterize the mechanical and wetting behaviors of liquid metals and their oxide layer. DSN measurements with the “air-indent” method have revealed new insights into the complex mechanical behavior of EGaIn droplets that are relevant to manipulate EGaIn at solid and flexible interfaces. The presence and chemistry of the oxide layer are directly related to the environment (air or various aqueous environments) and strongly affect the mechanical and interfacial adhesion responses of EGaIn. Droplets with a GaOOH oxide layer exhibit a weaker adhesion on solid substrates, whereas oxide-free droplets are more deformable and develop elongated capillary bridges at the EGaIn/solid substrate with higher adhesive forces. Depending on the magnitude of these interfacial adhesive forces, the EGaIn capillary bridges develop either positive or negative Gaussian curvatures, which result in positive or negative capillary pressures applying repulsive or attractive forces at the interface, respectively, with the former enhancing the mechanical stability of the capillary bridges. During both compressive and tensile loading, DSN also enabled one to detect microcracking and reformation of the oxide skin, a mechanism that is more obvious at lower strain rates, indicating that forming EGaIn junctions at higher strain rates should result in smoother EGaIn surfaces. Clear differences in interfacial adhesion and building up of EGaIn bridges (capillaries) were identified depending on the surface energy of the SAM adsorbed at the interface. The more polar OH-terminated SAMs resulted in the highest adhesive forces, whereas the nonpolar CH_3_-terminated SAMs gave the weakest interfacial adhesives forces. All of these factors affect the adhesion force of EGaI, which in turn directly affects the contact resistance. Our experiments have revealed a direct relation between adhesion forces and contact resistances, highlighting the importance of our work for applications of EGaIn in (molecular) electronics. The versatile interplay between surface energies and strain rates on the formation of capillary bridges or reformation dynamics of the GaO*_x_* layer revealed in this work will likely also be important in other areas of research including 3D printing of liquid metals, microwelding techniques based on liquid-metal droplets, and applications where EGaIn is continuously exposed to (repetitive) shearing and tensile forces.

## Experimental Section

### Materials and Chemicals

All of the solvents and chemicals were ordered from Sigma-Aldrich or Tokyo Chemicals Industry Co., Ltd. unless especially mentioned. All reagents were used directly as supplied unless mentioned otherwise. The deionized water was obtained from the Elga Purelab option-Q system. Silica gel (high-purity grade, pore size 60 Å, 40–63 μm particle size) was purchased from Sigma-Aldrich. The thiols HS-(CH_2_)_11_-CH_3_, HS-(CH_2_)_11_-OH, and HS-(CH_2_)_11_-NH_2_·HCl were purchased from Sigma-Aldrich with a purity of 99%. The HS-(CH_2_)_11_-NH_2_·HCl was neutralized and the HS-(CH_2_)_11_-NO_2_ was synthesized following previous procedures.^51^ The thiols were stored under the protection of N_2_ in a −50 °C freezer. We monitored the purity of the precursors once every two weeks using thin-layer chromatography (TLC). The Au and Ag (purity: 99.99%) were purchased from MOS Group Pte. Ltd. (Singapore). The ethanol (assay: 99.94% in V/V) was purchased from VWR Chemicals (France) and was dried over sodium ethoxide and distilled freshly for SAM incubation.

### Depth-Sensing Indentation/Air-Indent Method

Depth-sensing nanoindentation measurements were done using a Triboindenter TI-950 (Hysitron-Bruker, MN) equipped with an XZ-500 extended displacement stage allowing a vertical displacement of up to 500 μm. Prior to the DSN measurements, the tip was manually (using TriboScan software) positioned above the EGaIn sample at a distance of about 300 μm. The measurements were conducted using the “air-indent” mode, allowing us to run the measurements without any preapproach/contact on the sample surface. This enabled us to record the load–displacement curves during the tip approaching and its engagement to a pristine sample surface/oxide layer. Customized tungsten carbide (WC) tips with radii of 125 μm (for studies done in Figures 1 and 3) and 375 μm (Figure 4) were used. For the SAM experiments, the WC tips were Au-coated by sputtering for 60 s (*I* = 20 mA) to provide a 10 nm Au-coating. The experiments were carried out within 2 h after skimming the sample surfaces using a glass needle to provide fresh surfaces and minimize the effects of GaO*_x_* thickness on the measurements. We repeated the DSN measurements at least 5 times.

### Optical Microscopy

Optical micrographs (Figures 2 and 4) were captured using an EOS 700D camera equipped with an EF 100 macro lens (Canon) and an Axio Scope.A1 (ZEISS, Germany) optical microscope.

### Negligible Effect of Gravitational Force *F*_g_ on the Measured Attractive Forces

Considering the density of the EGaIn (6.25 g/mL, Sigma-Aldrich, Product ID: 495425) and the high resolution of the force transducer used in our DSN measurements (1 μN), we investigated the possible contribution of the gravitational force *F*_g_ acting on the EgAIn bridges. The extracted geometrical information of the capillary bridges measured using optical micrographs and indentation curves were used to construct and calculate the volume of the bridges. Our calculation revealed that even for a capillary bridge with a height of 100 μm (*V* ≈ 150,000 μm^3^), *F*_g_ does not exceed 0.1 μN, which is 3–4 orders of magnitude smaller than the recorded adhesion forces at the tip/EGaIn detachment. *F*_g_ can thus be ignored in the analysis based on eq 1.

### Comparative Surface Tensions for Different Tip/EGaIn Contact Regimes

According to eq 1 and the measured contact angles (Figure 2), the following parameters have been used for parametric calculation of surface tensions. For regime 1 (*r*_a_ > *r*_m_), we used (θ + ϕ) ≈ 50° (calculated form optical micrographs), 80 μm < *r*_c_ < 120 μm (for contact depths *h* < 50 μm measured in DSN measurements), *r*_c_ ≈ *r*_a_ > *r*_m_ (*r*_a_ = *r*_c_ – *r*_c_/10 and *r*_m_ = *r*_c_/2.5 (estimated from optical micrographs)), and *F*_ad_ = −25 μN (water) and −5μN (air) (measured from DSN experiments). For regime 2 (*r*_m_ > *r*_a_), we used (θ + ϕ) ≈ 64° (calculated form optical micrographs), 50 μm < *r*_c_ < 100 μm (for *h* < 50 μm, implemented in DSN measurements), *r*_m_ > *r*_a_ (*r*_a_ = *r*_c_ – *r*_c_/10) and *r*_m_ = 2.5 *r*_c_, (estimated from optical micrographs), and *F*_ad_ = −40 to −300 μN (NaOH).

### Metal Substrate Preparation and SAM Formation

The preparation of template-stripped Ag and Au substrates and SAM formation was the same as previously reported.^29^ The root-mean-square roughness values of the template-stripped Ag and Au were 0.6 ± 0.1 nm over an area of 4.0 × 4.0 μm^2^ and 0.3 ± 0.1 nm over an area of 1.0 × 1.0 μm^2^ (the error bars were the standard deviation from three measurements from three substrates). The SAMs formed on the template-stripped Ag and Au surfaces and WC tips with Au coatings in ethanolic solutions with 3 mM of the respective SAM precursor. The SAMs were formed over a period of time of 3 h before they were taken out of the solutions, washed with ethanol, and finally dried in a stream of N_2_.

### Electrical *J*(V) Characterization

The EGaIn setup and collection of *J*(V) curves of the junctions were the same as in a previous report.^45^ We used a Keithley 6340 source meter and a Labview 2010 to apply voltage and collect the *J*(V) raw data. Briefly, for each type of SAM on the template-stripped surfaces, we formed junctions with cone-shaped tips of EGaIn. We collected *J*(V) curves from ∼20 junctions from three substrates, and for each junction, ∼20 traces were collected. We averaged the *J*(V) curves by Gaussian-fitting the logarithm values of the absolute current density for each measured voltage *V* to obtain the Gaussian log-average, <log_10_|*J*|>_G_, with Gaussian log-standard deviation of *σ*_log,G_, which are shown in Figures S3 and S4 for *V* = +1.0 and −1.0 V. The < log_10_|*J*|>_G_ as a function of *V* curves is shown in Figure S2.

### Impedance Characterization

The impedance spectroscopy characterization was carried out following a previous report with the EGaIn confined in microchannels of polydimethylsiloxane (PDMS) as the top electrode.^46^ The impedance was measured with 30 mV of sinusoidal perturbation across the frequency range of 10^6^–10^2^ Hz, which is shown in Figure S7, with a Solartron 1296 Dielectric Interface and a Solartron SI 1260 Impedance/Gain-Phase Analyzer. We used the SMaRT v3.2.1 to record and the ZView to fit the data. The residual plots from the fits and the Kramers–Kronig (KK) transformations are shown in Figures S5 and S6, and they demonstrate that the data of the equivalent circuit fitted well to our data within the given error from the KK plots: residuals of the fits (χ_fit_^2^) and KK (χ_KK_^2^) show random noise within 10% of the raw data with values of χ_fit_^2^ and χ_KK_^2^ ranging from 0.0004 to 0.0017. Note, we used the same equivalent circuit (see the inset in Figure S7a) that is known to represent our junctions well as previously reported^46,47^ (all fitting results are given in Table S2).

### Contact Angle Measurements

We used the static contact angle method to determine the surface energy of SAMs using the DataPhysics Instrument with the model OCA 25 (Filderstadt, Germany) to measure, fit, and analyze the results. The OWRK (Owens, Wendt, Rabel, and Kaelble) model^48−50^ is a standard method to calculate the surface energy of a solid using the contact angle of liquids. The surface free energy γ_s_ is separated into a dispersive part (γ_s_^D^) and a polar part (γ_s_^P^). At least two kinds of liquids are needed, and at least one must have a polar part >0. Here, we used deionized water and diiodemethane (DIM) to obtain the polar (γ_s_^P^) and dispersive (γ_s_^D^) forces on adhesion using the OWRK method. The DIM has no polar part, γ_l_^P^ = 0. The γ_s_^P^ and γ_s_^D^ are obtained from γ_s_ = γ_sl_ + γ_l_ × cos θ and . Figures S8–S12 show the optical photographs along with fits of the drops of H_2_O and DIM of the SAMs on Ag and Au from which we derived the contact angles tabulated in Tables S3 and S4 and the driven surface free energies plotted in Figure S12. The results for the EGaIn droplets on SAMs are shown in Figures S13 and S14.

### Data Plotting

OriginPro 2021 was used for plotting of the data.

## Supporting Information Available

Adhesion forces, electrical *J*(V), impedance, and contact angle results, and associated tables used in the paper (PDF)

## Notes

The data sets generated and/or analyzed during the current study are available from the corresponding author upon reasonable request.
