# Supplementary material for: Interplay between Interfacial Energy, Contact Mechanics, and Capillary Forces in EGaIn Droplets
Source: ACS Appl Mater Interfaces. 2022 Jun 1;14(24):28074–84. doi: 10.1021/acsami.2c04043 (PMC9227710; doi:10.1021/acsami.2c04043)
Supplement: Supplementary file 1 — am2c04043_si_001.pdf [file am2c04043_si_001.pdf]

Supporting Information

Interplay between Interfacial Energy, Contact Mechanics and Capillary Forces in EGaIn Droplets

Shahrouz Amini[1,2,#,], Xiaoping Chen[3,4,#], Jia Qing Isaiah Chua[2], Jinq Shi Tee[4], Christian A. Nijhuis[4,5,6,*], and Ali Miserez[2,7,*]*

[1] Department of Biomaterials, Max Planck Institute of Colloids and Interfaces, 14476 Potsdam, Germany

[2] Biological and Biomimetic Materials Laboratory, Center for Sustainable Materials (SusMat), School of Materials Science and Engineering, Nanyang Technological University (NTU), 50 Nanyang Avenue, 639798, Singapore

[3] Department of Chemistry and Environment Science, Fujian Province Key Laboratory of Modern Analytical Science and Separation Technology, Minnan Normal University, Zhangzhou 363000, China

[4] Department of Chemistry, National University of Singapore, 3 Science Drive 3, Singapore 117543, Singapore

[5] Centre for Advanced 2D Materials and Graphene Research Centre, National University of Singapore, 6 Science Drive 2, Singapore 117546, Singapore

[6] Hybrid Materials for Opto-Electronics Group, Department of Molecules and Materials, MESA+ Institute for Nanotechnology and Molecules Centre, Faculty of Science and Technology, University of Twente, 7500 AE Enschede, The Netherlands

[7] School of Biological Sciences, Nanyang Technological University (NTU), 60 Nanyang Drive, Singapore 637551, Singapore

[#] These authors contributed equally to the study.

* Corresponding authors: Shahrouz.Amini@mpikg.mpg.de; ali.miserez@ntu.edu.sg; c.a.nijhuis@utwente.nl

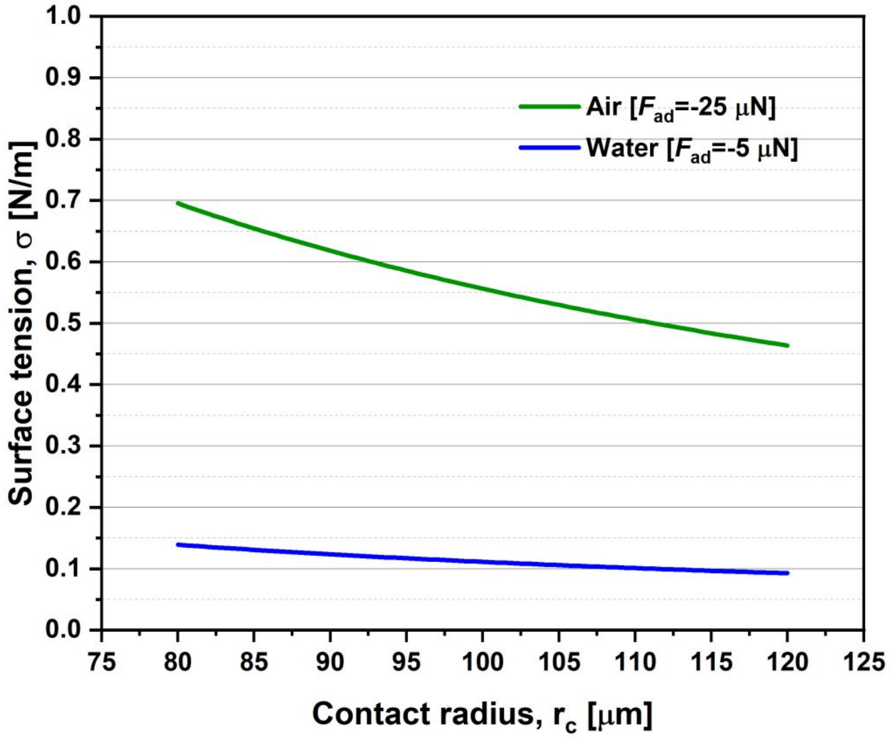

Figure S1. γ *vs.* r_c plots at adhesion forces F_{ad} corresponding to measurements in air and water.

Figures S2-4 show the Gaussian log-averaged J(V) curves, <$\log_{10}|J|$>$_G$, with error bars of $\sigma_{log,G}$, and the histograms of the $\log_{10}|J|$ at ±1.0 V for the junctions of different types of SAMs.

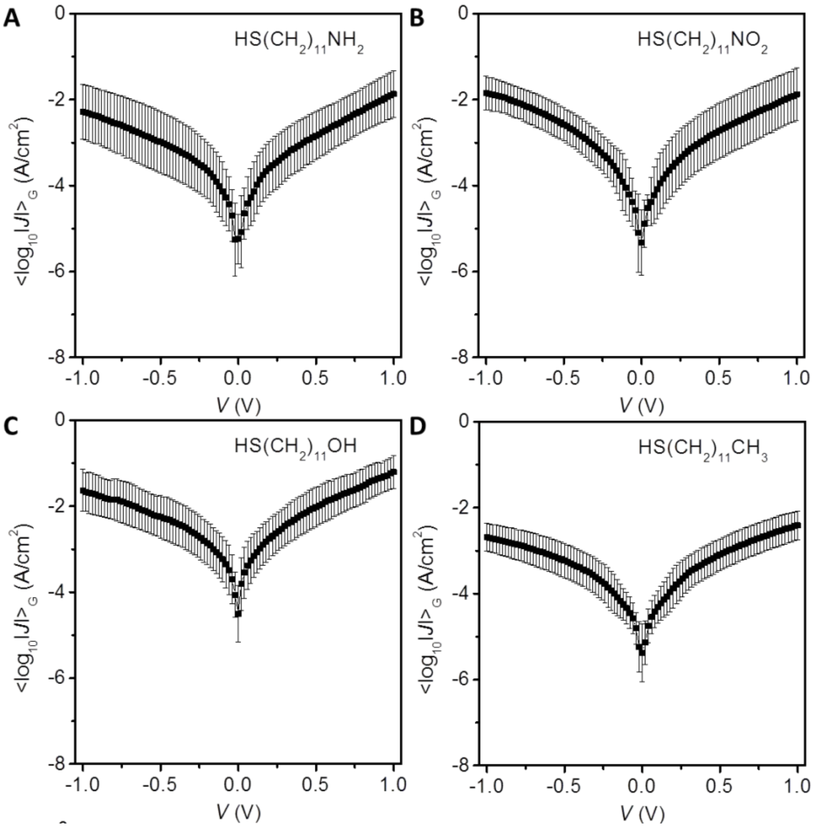

Figure S2. <$\log_{10}|J|$>$_G$ *vs* V(V) for Ag-S$(CH_2)_n$X//GaO_x/EGaIn junctions of (**A**) HS$(CH_2)_{11}NH_2$, (**B**) HS$(CH_2)_{11}NO_2$, (**C**) HS$(CH_2)_{11}$OH, and (**D**) HS$(CH_2)_{11}CH_3$ SAMs with error bar of $\sigma_{log,G}$ from Gaussian fit.

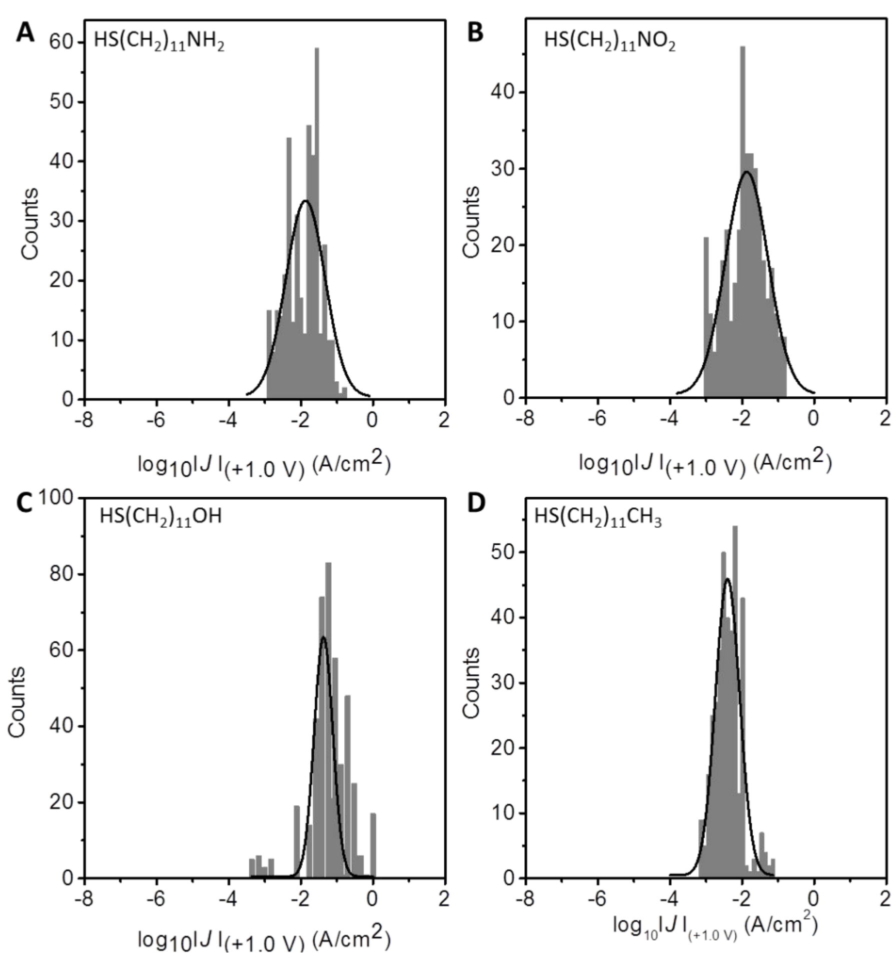

Figure S3. Histograms of $\log_{10}|J|$ for Ag-S$(CH_2)_n$X//GaO_x/EGaIn junctions of (**A**) $HS(CH_2)_{11}NH_2$, (**B**) $HS(CH_2)_{11}NO_2$, (**C**) $HS(CH_2)_{11}OH$, and (**D**) $HS(CH_2)_{11}CH_3$ SAMs at +1.0 V with Gaussian fit (black lines).

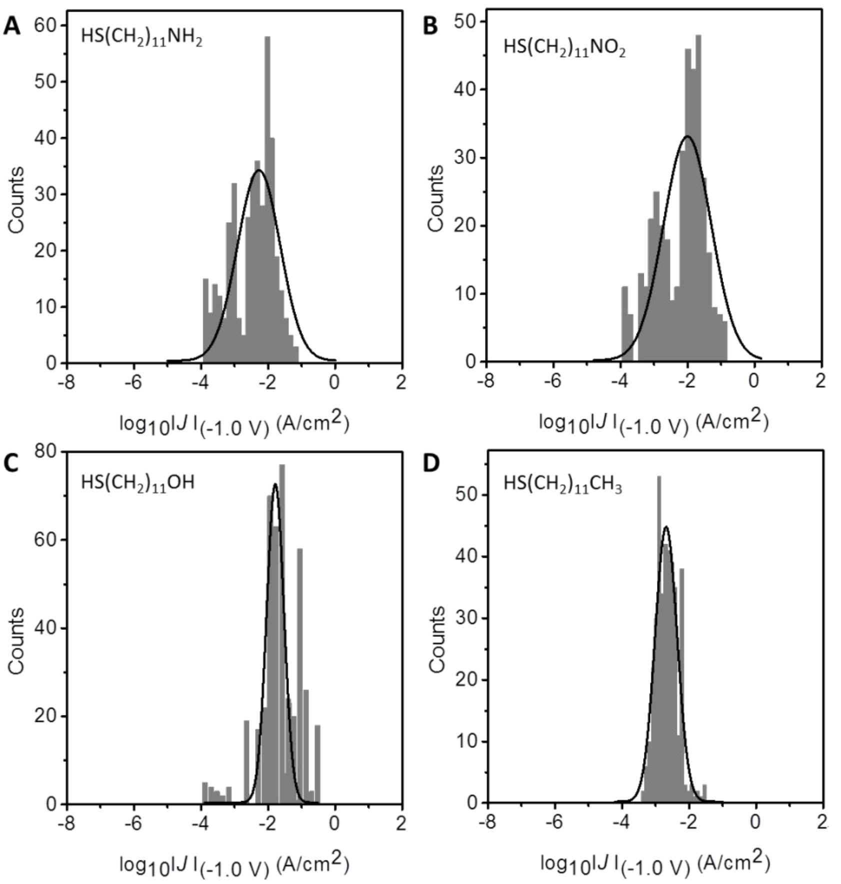

Figure S4. Histograms of $\log_{10}|J|$ for Ag-S$(CH_2)_n$X//GaO_x/EGaIn junctions of (**A**) HS$(CH_2)_{11}NH_2$, (**B**) HS$(CH_2)_{11}NO_2$, (**C**) HS$(CH_2)_{11}$OH, and (**D**) HS$(CH_2)_{11}CH_3$ SAMs at -1.0 V with Gaussian fit (black lines).

Figure S5-7 show the residual plots, the Bode, Nyquist, and phase angle vs frequency plots of the molecular junctions.

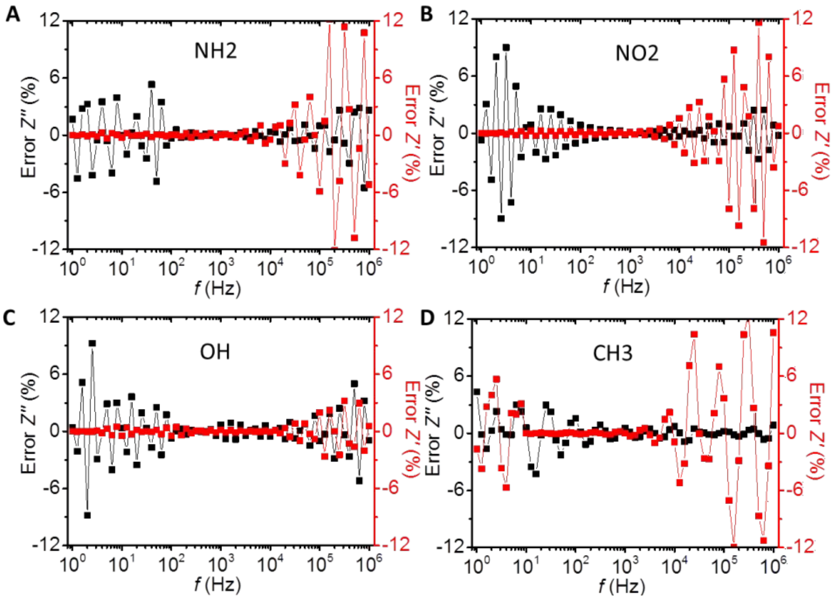

Figure S5. The residual plots of the junctions of (**A**) $HS(CH_2)_{11}NH_2$, (**B**) $HS(CH_2)_{11}NO_2$, (**C**) $HS(CH_2)_{11}OH$, and (**D**) $HS(CH_2)_{11}CH_3$ SAMs from impedance fit.

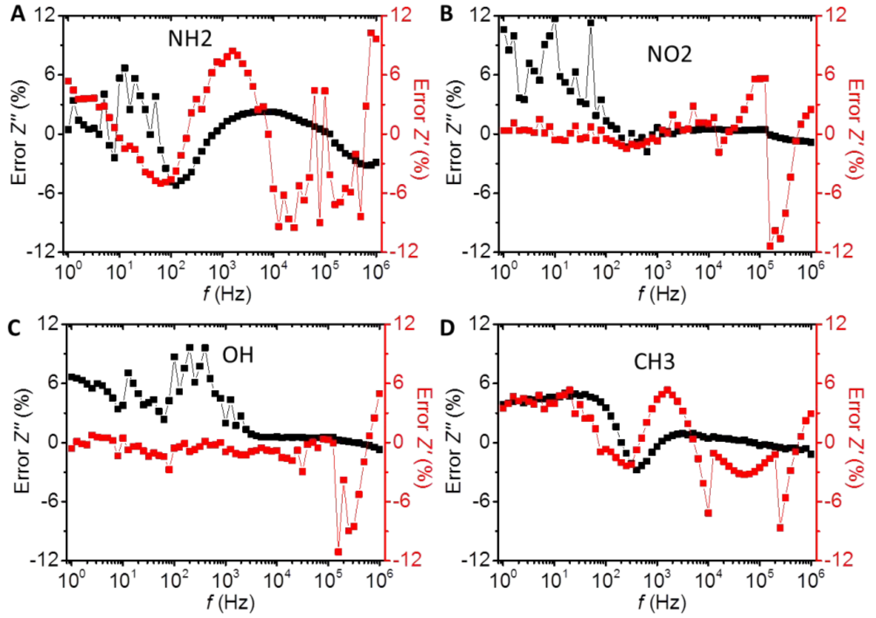

Figure S6. The KK residual plots of the junctions of (**A**) HS$(CH_2)_{11}NH_2$, (**B**) HS$(CH_2)_{11}NO_2$, (**C**) HS$(CH_2)_{11}$OH, and (**D**) HS$(CH_2)_{11}CH_3$ SAMs from impedance fit.

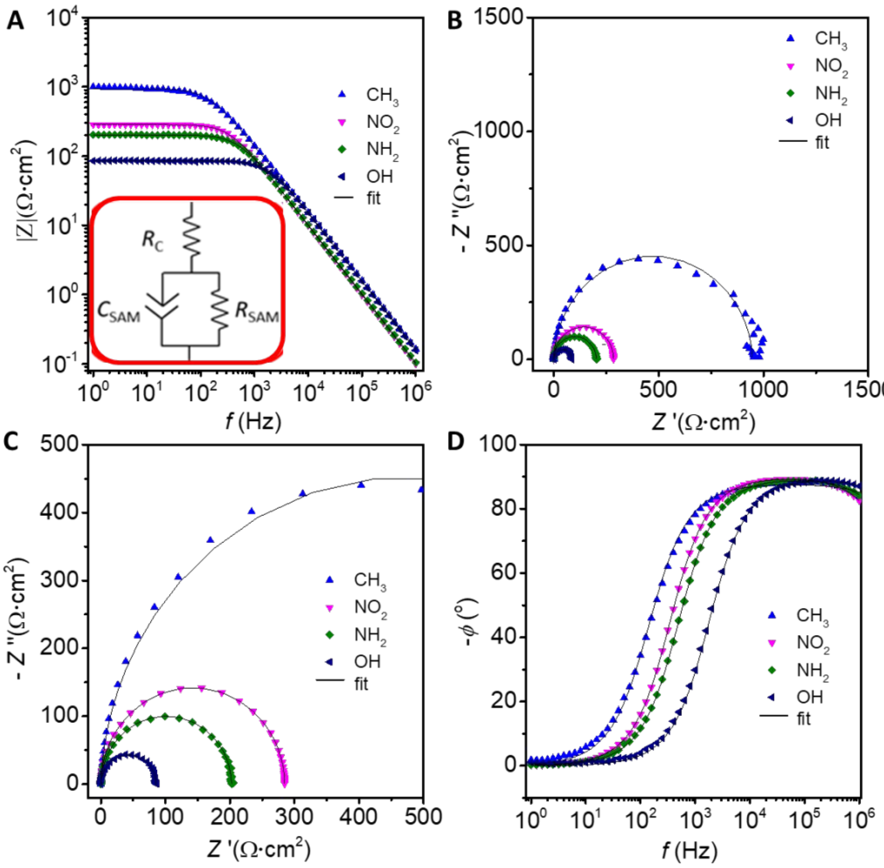

Figure S7. Representative Bode plots (**A**), inset is the equivalent circuit used to fit the data; Nyquist plots (**B**), the zoom in of Nyquist plots (**C**); and phase angle vs frequency plots (**D**) at 0 V for Ag-S$(CH_2)_n$X//GaO_x/EGaIn junctions. The black solid lines are fits of the data to the equivalent circuit shown in panel A.

Figures S8-12 show the real contact angles of the SAMs on Ag and Au using H_2O and DIM and the driven surface free energies.

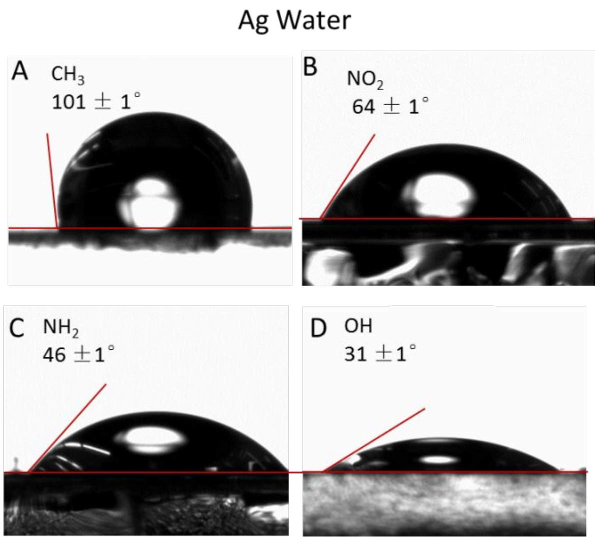

Figure S8. Represent photos of contact angles of a water drop on (**A**) $HS(CH_2)_{11}CH_3$, (**B**) $HS(CH_2)_{11}NO_2$, (**C**) $HS(CH_2)_{11}NH_2$, and (**D**) $HS(CH_2)_{11}OH$ SAMs on Ag.

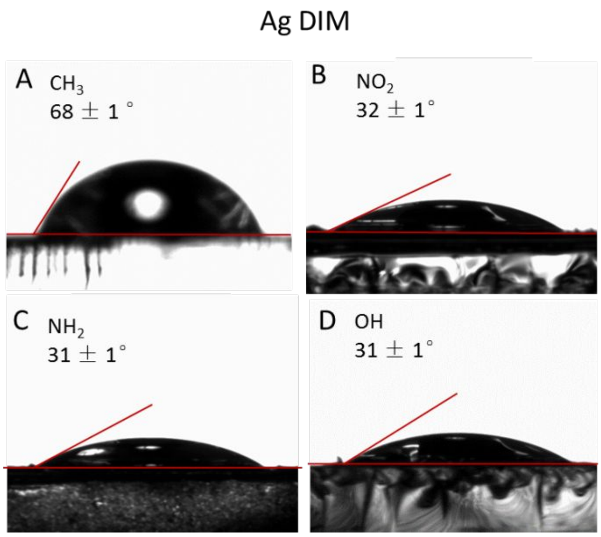

Figure S9. Represent photos of contact angles of a DIM drop on (**A**) $HS(CH_2)_{11}CH_3$, (**B**) $HS(CH_2)_{11}NO_2$, (**C**) $HS(CH_2)_{11}NH_2$, and (**D**) $HS(CH_2)_{11}OH$ SAMs on Ag.

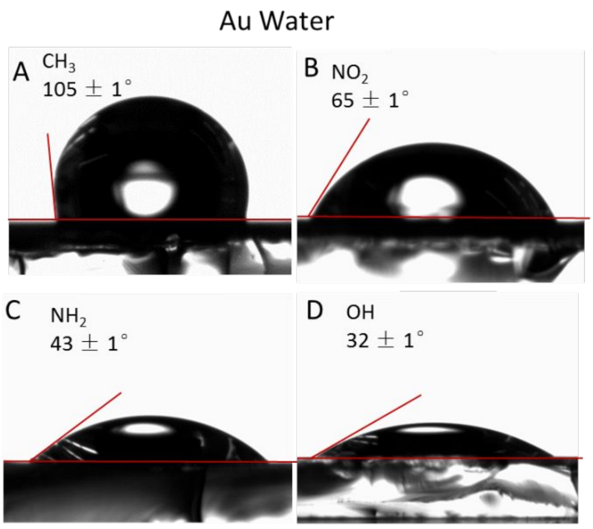

Figure S10. Represent photos of contact angles of a water drop on (**A**) $HS(CH_2)_{11}CH_3$, (**B**) $HS(CH_2)_{11}NO_2$, (**C**) $HS(CH_2)_{11}NH_2$, and (**D**) $HS(CH_2)_{11}OH$ SAMs on Au.

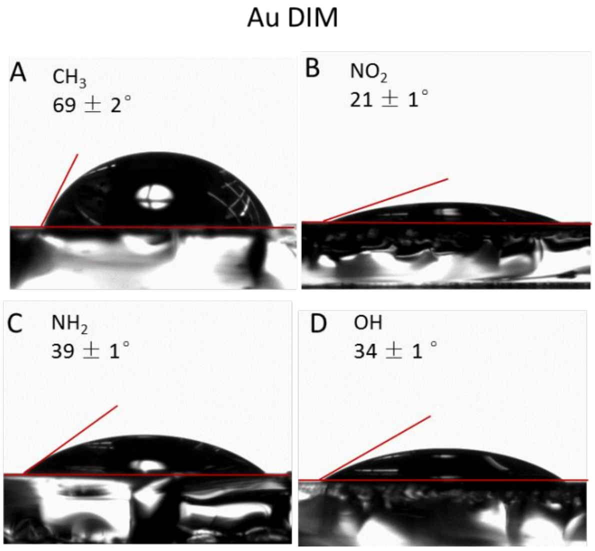

Figure S11. Represent photos of contact angles of a DIM drop on (**A**) $HS(CH_2)_{11}CH_3$, (**B**) $HS(CH_2)_{11}NO_2$, (**C**) $HS(CH_2)_{11}NH_2$, and (**D**) $HS(CH_2)_{11}OH$ SAMs on Au.

.

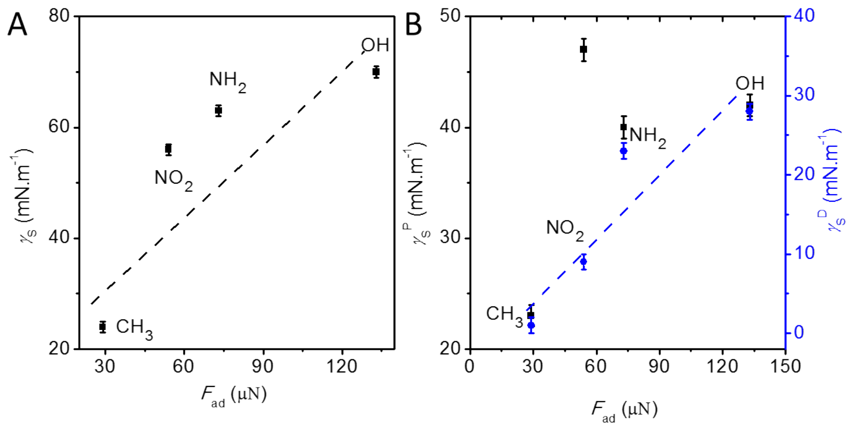

Figure 12. (**A**) Surface free energy and (**B**) polar (black squares) and dispersive forces (blue circles) vs F_{ad} of SAMs on Au from contact angle measurements. The error bars represent the standard deviation from three different sets of measurements of the contact angles. The dashed lines are guides to the eye.

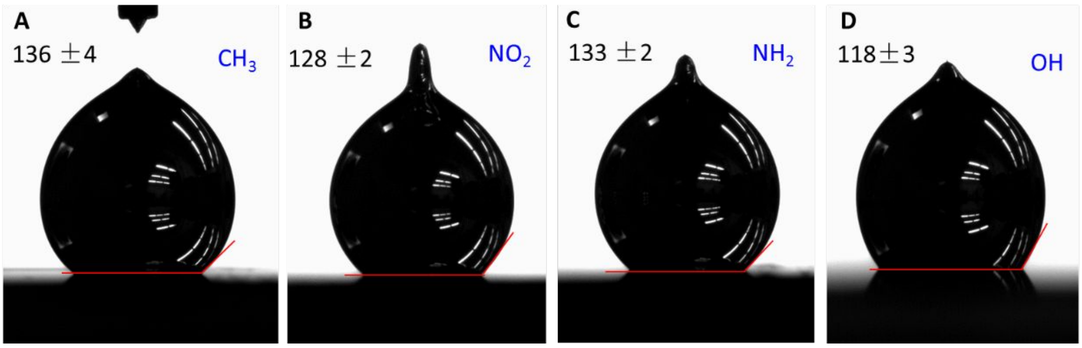

Figure S13. Represent photos of contact angles of EGaIn drops on (**A**) $HS(CH_2)_{11}CH_3$, (**B**) $HS(CH_2)_{11}NO_2$, (**C**) $HS(CH_2)_{11}NH_2$, and (**D**) $HS(CH_2)_{11}OH$ SAMs on Ag.

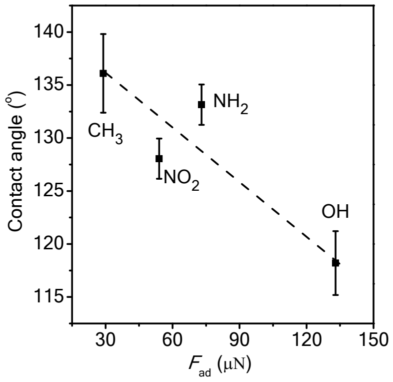

Figure S14. Contact angles of EGaIn drops on SAMs vs F_{ad} from AFM measurements.

Tables

Table S1. Summary of J(V) results of junctions of Ag-S(CH_2)$_n$X//GaO_x/EGaIn at ±1.0 V.

Terminal	No. of junctions	No. of unstable or shorted junctions	Yield (%)	No. of traces	$<\log_{10}\vert J\vert>_{G(+1.0\ V)} \pm \sigma_{\log,G}$ (A/cm^2)	$<\log_{10}\vert J\vert>_{G(-1.0\ V)} \pm \sigma_{\log,G}$ (A/cm^2)
-NH_2	22	2	91	396	-1.9 ± 0.5	-2.3 ± 0.6
-NO_2	23	4	83	378	-1.9 ± 0.6	-2.0 ± 0.7
-OH	26	3	88	453	-1.4 ± 0.3	-1.8 ± 0.3
-CH_3	21	2	90	380	-2.4 ± 0.3	-2.7 ± 0.3

Table S2. Summary of impedance results of junctions of Ag-S(CH_2)$_n$X//GaO_x/EGaIn at ±1.0 V.

Terminal	$R_{SAM} \pm \sigma$ (Ω/cm^2)	$R_C \pm \sigma$ (mΩ/cm^2)	$C_{SAM} \pm \sigma$ (μF.cm^2)	n_{CPE}
-NH_2	(0.9 ± 0.1) × 10^3	7.5 ± 0.3	1.49 ± 0.08	0.996 ± 0.008
-NO_2	(0.3 ± 0.1) × 10^3	9.1 ± 0.4	1.69 ± 0.04	0.992 ± 0.006
-OH	(0.07 ± 0.01) × 10^3	6.7 ± 0.7	1.48 ± 0.21	0.994 ± 0.002
-CH_3	(1.3 ± 0.4) × 10^3	10.8 ± 0.8	1.48 ± 0.14	0.995 ± 0.010

Table S3. Summary of contact angle results of junctions of Ag-S(CH_2)$_n$X.

Terminal	θ_{H2O} (°)	θ_{DIM} (°)	γ_s(mN.m^{-1})	γ_s^P(mN.m^{-1})	γ_s^D (mN.m^{-1})
-NH_2	46 ± 1	31 ± 1	63 ± 4	43 ± 1	19 ± 3
-NO_2	64 ± 1	32 ± 1	53 ± 1	43 ± 1	10 ± 1
-OH	31 ± 1	31 ± 1	71 ± 1	44 ± 1	28 ± 1
-CH_3	101 ± 1	68 ± 1	25 ± 1	24 ± 1	1 ± 1

Table S4. Summary of contact angle results of junctions of Au-S(CH_2)$_n$X.

Terminal	θ_{H2O} (°)	θ_{DIM} (°)	γ_s(mN.m^{-1})	γ_s^P(mN.m^{-1})	γ_s^D (mN.m^{-1})
-NH_2	43 ± 1	39 ± 1	63 ± 1	40 ± 1	23 ± 1
-NO_2	65 ± 1	21 ± 1	56 ± 1	47 ± 1	9 ± 1
-OH	32 ± 1	34 ± 1	70 ± 1	42 ± 1	28 ± 1
-CH_3	105 ±1	69 ± 2	24 ± 1	23 ± 1	1 ± 1
